# A meta-ethnography of language challenges in midwifery care

**DOI:** 10.18332/ejm/147994

**Published:** 2022-07-05

**Authors:** Victoria L. Sellevold, Lene L. Hamre, Terese E. Bondas

**Affiliations:** 1 Faculty of Health Sciences, University of Stavanger, Stavanger, Norway; 2 Childbearing - Qualitative Research Network, Faculty of Health Sciences, University of Stavanger, Stavanger, Norway

**Keywords:** immigration, communication, language, meta-ethnography, foreign women, qualitative review

## Abstract

**INTRODUCTION:**

The aim was to synthesize qualitative studies that shed light on immigrant women’s own experiences regarding language challenges during pregnancy, birth and the postpartum period in a new country. Wikberg’s theory of intercultural caring was chosen as the theoretical perspective.

**METHODS:**

This meta-ethnography was conducted in accordance with Noblit and Hare, an interpretative qualitative review approach in seven phases, based on inclusion and exclusion criteria, and a systematic and manual search yielding a total of 1253 articles. Seven articles were included after a quality appraisal using the CASP instrument, and the process is shown in a PRISMA chart. The 142 women from the studies represented 42 different nationalities and were residents in 6 different host countries.

**RESULTS:**

A synthesis model illuminates the immigrant women’s experiences of language challenges. ‘To comprehend and to be understood as a unique person’ emerged as the overarching theme, based on the three analogous themes: ‘a desire to be met with respectful understanding’, ‘ethical and accurate interpreting’, and ‘caring midwives and new relationships'. The fourth opposite theme, ‘violations and feelings of inferiority’ describes the immigrant women’s experiences when the language challenges remained.

**CONCLUSIONS:**

Midwives should be aware of language challenges in pregnancy, birth and postpartum care to minimize the risk of violations and unnecessary suffering related to care. The synthesis model can serve as a guide for respectful understanding, ethical and accurate interpreting, and caring, in accordance with Wikberg’s dimensions of intercultural caring: universality, context, culture, and the needs and wishes of the immigrant women.

## INTRODUCTION

According to recent data, 3.5% of the world’s population are international migrants, and this seems to be a rising trend^1,2^. The number of refugees has not been this high since World War II^3-7^. As of 1 January 2020, 24 million non-European citizens were living in a country in Europe, and in 2019, 2.7 million people immigrated to a European country; 54% of these immigrants were men and 46% were women, with a median age of 29.2 years^2^. One can assume this includes many women of childbearing age. A large number of these women speak a foreign language and not the host country’s language, and they might have little to no knowledge of the country’s second language. For this reason, it may be appropriate to think that midwives should possess knowledge of what these immigrant women may experience in relation to midwifery care throughout pregnancy, birth, and the postnatal period.

There is a trend showing worse outcomes for immigrant women compared to native women when it comes to pregnancy, childbirth and the postnatal period. For example, there is increased maternal morbidity and mortality, and increased occurrence of serious illness and mental health issues, such as postpartum depression. There is also a higher risk of premature birth, stillbirth and genital injuries, and otherwise poorer follow-up and quality of care. Possible explanatory factors for these varying outcomes in some of these women include, but are not limited to, genetic and biological factors, a higher incidence of certain diseases in their countries of origin (such as HIV and tuberculosis), and social factors such as lower education and socioeconomic status. Misconceptions among immigrant women and midwives may occur when there are language challenges. Misconceptions may be indirectly related to differences in cultural concepts and the acceptance of care, and may be a direct result of the lack of interpreting services and problems in the healthcare system, such as a lack of access to care, and social security issues^8^.

The theoretical perspective chosen, which is Wikberg’s theory on intercultural caring related to pregnancy, childbirth, and the postnatal period, consists of the four dimensions of universal, cultural, contextual, and unique caring^9-13^. When intercultural caring is missing, it may be replaced by inhuman and uncaring encounters that, from the women’s perspectives, may be experienced as violation of dignity and nonchalance. The language challenges may result in the insufficient provision of care and support, which is conceptualized as suffering related to care, and this may cause a burden for the woman^14,15^. There is a need to illuminate immigrant women’s own experiences regarding language challenges during pregnancy, birth, and the postnatal period, by integrating knowledge from previous qualitative studies in order to increase midwives’ knowledge concerning communication with immigrant women in order to ensure that they can provide the best midwifery care.

## METHODS

### Research aim

The aim of this meta-ethnography was to integrate and synthesize previous qualitative research on immigrant women’s experiences of language challenges during pregnancy, birth and the postpartum period.

### Research design

It was decided that the most appropriate approach was to choose an interpretative meta-ethnography, as we focused on the women’s own perspectives as described in previous qualitative studies. We could, therefore, in the translation and interpreting, explore new meanings in already existing qualitative findings. Malterud^16^ and Bondas and Hall^17^ are among those who argue for depth and strength in findings across studies using this method. We used the eMERGe checklist (Table 1) for guidance^18^ throughout the study to ensure adequate reporting and transparency, and to increase the credibility of the findings and the process. This checklist is therefore suitable for our meta-ethnographic approach.

**Table 1:** The eMERGe checklist for meta-ethnography

		*PAGES*
**Phase 1** **Selecting meta-ethnography and getting started**	**1. Rationale and context for the meta-ethnography** Describe the gap in research or knowledge to be filled by the meta-ethnography, and the wider context of the meta-ethnography.	1–2
	**2. Aim(s) of the meta-ethnography** Describe the meta-ethnography aim(s).	2
	**3. Focus of the meta-ethnography** Describe the meta-ethnography review question(s) (or objectives).	1–2
	**4. Rationale for using meta-ethnography** Explain why meta-ethnography was considered the most appropriate qualitative synthesis methodology.	2
**Phase 2** **Deciding what is relevant**	**5. Search strategy** Describe the rationale for the literature search strategy.	3
	**6. Search processes** Describe how the literature searching was carried out and by whom.	2–3
	**7. Selecting primary studies** Describe the process of study screening and selection, and who was involved.	3
	**8. Outcome of study selection** Describe the results of study searches and screening.	3
**Phase 3** **Reading included studies**	**9. Reading and data extraction approach** Describe the reading and data extraction method and processes.	3–4
	**10. Presenting characteristics of included studies** Describe characteristics of the included studies.	3, Table 3
**Phase 4** **Determining how studies are related**	**11. Process for determining how studies are related** Describe the methods and processes for determining how the included studies are related: Which aspects of studies were compared AND How the studies were compared.	3–4
	**12. Outcome of relating studies** Describe how studies relate to each other.	3–4
**Phase 5 Translating studies into one another**	**13. Process of translating studies** Describe the methods of translation: Describe steps taken to preserve the context and meaning of the relationships between concepts within and across studies Describe how the reciprocal and refutational translations were conducted Describe how potential alternative interpretations or explanations were considered in the translations.	3-4
	**14. Outcome of translation** Describe the interpretive findings of the translation.	4–13
**Phase 6 Synthesizing translations**	**15. Synthesis process** Describe the methods used to develop overarching concepts (‘synthesized translations’). Describe how potential alternative interpretations or explanations were considered in the synthesis.	8–9
	**16. Outcome of synthesis process** Describe the new theory, conceptual framework, model, configuration, or interpretation of data developed from the synthesis.	8–9, Figure 2
**Phase 7 Expressing the synthesis**	**17. Summary of findings** Summarize the main interpretive findings of the translation and synthesis and compare them to existing literature.	10–13
	**18. Strengths and limitations** Reflect on and describe the strengths and limitations of the synthesis: for example, describe how the synthesis findings were influenced by the nature of the included studies and how the meta-ethnography was conducted.	3,13
	**19. Recommendations and conclusions** Describe the implications of the synthesis.	13–14

### Inclusion criteria

The inclusion criteria were that the relevant studies should be qualitative and use the women’s perspective, be published in a Scandinavian language or in English, and be based on an original peer-reviewed qualitative research study. The exclusion criteria applied to quantitative studies and qualitative studies published earlier than 2010. We wanted new and updated knowledge and perspectives from increasingly multicultural and global societies.

### Systematic search

**Figure 1:**
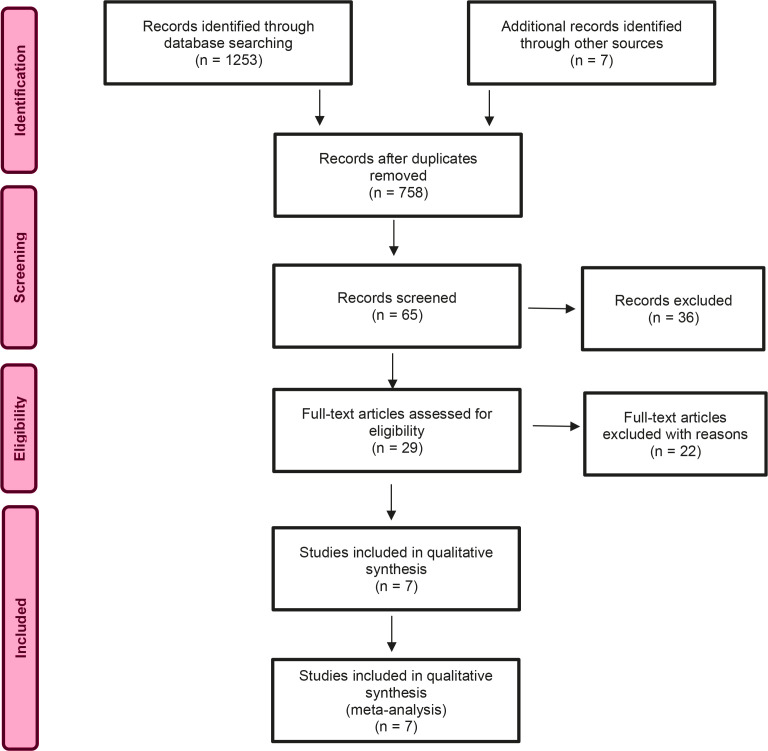
PRISMA flowchart

To collect data, the first and second author used three relevant search databases – the British Nursing Index, CINAHL, and Medline, on three different dates between May and October 2020. In the process, we also completed reference chain searches and manual searches^18^. The search process and article selection are documented in a PRISMA flow chart (Figure 1)^19^. Keywords used during the literature searches were: childbirth, immigrant, midwife, communication, language, language barrier, woman, and maternity care. The searches included synonyms and variations/truncations.

A systematic and manual search yielded a total of 1253 articles. After reading and selection, the critical appraisal of qualitative research, CASP^20^ was used to assess the usefulness and quality of the seven included studies (Table 2). The seven studies were all considered to have high quality.

**Table 2:** CASP - quality assessment of the included studies

*Authors*	*Clear aims*	*Appropriate methodology*	*Appropriate design*	*Appropriate recruitment strategy*	*Appropriate data collection*	*Adequate consideration on relationship between researcher and participant*	*Ethical considerations*	*Rigorous data analysis*	*Clear statement of findings*	*The value of the research*
Crowther and Lau^22^ (2019)	Y	Y	Y	Y	Y	Y	Y	Y	Y	Y
Sami et al.^24^ (2019)	Y	Y	Y	Y	Y	Y	Y	Y	Y	Y
Robertrson^25^ (2015)	Y	Y	Y	Y	Y	Y	Y	Y	Y	Y
Viken et al.^23^ (2015)	Y	Y	Y	Y	Y	Y	Y	Y	Y	Y
Higginbottom et al.^26^ (2016)	Y	Y	Y	Y	Y	Y	Y	Y	Y	Y
Wikberg et al.^12^ (2012)	Y	Y	Y	Y	Y	Y	Y	Y	Y	Y
Origlia-Ikhilor et al.^27^ (2019)	Y	Y	Y	Y	Y	Y	Y	Y	Y	Y

Y: yes.

The articles that were read in full text and/or as abstracts, but were not included in the study, were articles that contained little to nothing about language challenges in the midwifery context. Other excluded studies were articles that dealt with specific interventions such as doula or digital services, or where the perspective was not the women’s own.

We also drew a table with characteristics of the included studies, so that we could easily have an overview, and could describe and analyze the studies in the translation process (Table 3).

**Table 3:** Characteristics of the included studies

*Study*	*Study design and aim(s) of study*	*Participants*	*Context*	*Method of data collection and analysis*	*Key findings*
Crowther and Lau^22^ (2019) Scotland	Qualitative descriptive study Aim: to explore Polish migrant women’s experiences of language and communication concerns when accessing UK maternity services.	9 immigrants from Poland	Local maternity services in Scotland	Semi-structured interview Interviews in Polish and English according to the women’s choice.	Three descriptive themes: Communication and understanding, Relationships matter, and Values and expectations Seven subthemes
Sami et al.^24^ (2019) Switzerland	Qualitative exploratory study Aim: to describe the experiences of migrant women with pregnancy and maternity services at two main hospitals in Geneva and Zurich and to identify specific barriers for healthcare accessibility.	Six focus groups including 33 women in total	Two main hospitals in Geneva and Zurich	Focus groups using semi-structured questions developed by both researchers, one of them conducting the interviews, and the other observing.	Positive experiences included not only the availability of maternity services, especially during emergency situations and the postpartum period, but also the availability of specific maternity services for undocumented migrants in Geneva. Negative experiences were classified into either personal or structural barriers. On the personal level, the main barriers were a lack of social support and a lack of health literacy, whereas the main themes on the structural level were language barriers and a lack of information.
Robertrson^25^ (2015) Sweden	Qualitative descriptive method Aim: To analyze the reflections of women on how their experiences of migration and resettlement to Sweden influenced their health and healthcare needs during childbearing in Sweden.	25 women from 17 countries	Swedish antenatal care	Focus-group discussions, pair interviews and individual interviews	The hardships of migration, resettlement, and constraints in the daily life made the women feel overstrained, tense, and isolated. Being treated as a stranger and ignored or rejected in healthcare encounters was devaluing and discriminating. The women stressed that they felt stronger and had fewer complications during pregnancy and labor when they were ‘taken seriously’ and felt that they had a confident, caring relationship with caregivers/midwives. This, therefore, enabled the women to boost their sense of self, and to recognize their capabilities, as well as their ‘embodied knowledge’.
Viken et al.^23^ (2015) Norway	Qualitative exploratory, design with a hermeneutic approach Aim: To explore the maternal health coping strategies of migrant women in Norway.	17 immigrant women	Norwegian antennal care	Semi-structured interviews	One overall theme is as follows: keeping original traditions while at the same time being willing to integrate into Norwegian society, and four themes emerged: balancing their sense of belongingness; seeking information and support from healthcare professionals; being open to new opportunities; and focusing on feeling safe in the new country.
Higginbottom et al.^26^ (2016) Canada	Ethnographic research design Aim: To generate new understanding of the processes that perpetuate immigrant disadvantages in maternity healthcare, and devise potential interventions that might improve maternity experiences and outcomes for immigrant women in Canada.	86 participants: 34 immigrants 29 healthcare workers 26 stakeholders	Canadian maternity healthcare	Semi-structured individual and focus group interviews	The findings indicate that communication difficulties, lack of information, lack of social support (isolation), cultural beliefs, inadequate healthcare services, and cost of medicine/services represent potential barriers to the access to and navigation of maternity services by immigrant women in Canada.
Wikberg et al.^12^ (2012) Finland	Descriptive and interpretive ethnography Aim: To describe and interpret the perceptions and experiences of caring of immigrant new mothers from an intercultural perspective in maternity care in Finland.	17 immigrant mothers from 12 countries	Finnish antenatal care	Interviews, observations, and field notes were analyzed and interpreted.	Four patterns: There were differences between the expectations of the mothers and their Finnish maternity care experience of caring. Caring was related to the changing culture. Finnish maternity care traditions were sometimes imposed on the immigrant new mothers, which likewise influenced caring. However, the female nurse was seen as a professional friend, and the conflicts encountered were resolved, which in turn promoted caring.
Origlia-Ikhilor et al.^27^ (2019) Switzerland	Qualitative explorative study Aim: To describe communication barriers faced by allophone migrant women in maternity care provision from the perspectives of migrant women, healthcare professionals, and intercultural interpreters.	10 immigrant women 22 healthcare workers 4 interpreters	German speaking part of Switzerland	Focus group discussions and problem-centered interviews single or in pairs in the women’s own language. Researchers observed using an interpreter.	The analysis revealed three main themes: The challenge of understanding each other’s world, communication breakdowns, and imposed health services. Without interpretation communication was reduced to a bare minimum and thus in- sufficient to adequately inform women about treatment and address their expectations and needs.

### Analysis and synthesis

For the analysis process, we used Noblit and Hare’s^21^ seven-phase method for meta-ethnography. The first and second author performed the data extraction from the primary studies separately, in order to strengthen validity. This was done by printing all the articles and highlighting what we perceived as meaning units in the findings that corresponded to the aim in the findings; these were then checked and discussed with the third author. We also pre-recorded our preconceptions and viewed them in the light of the actual findings. Our pre-recorded pre-understanding worked as a tool through the analysis process, which ensured that findings that corresponded to the pre-understanding were not included unless we were certain of their objective relevance. The authors did not maintain a dialogue in the preconception writing process, as we did not want to influence each other. The synthesis was conducted using a matrix, wherein we organized metaphors and themes, beginning with an index study that contained rich data^22^. We entered all the studies and translated their content based on an assumption of analogy or similarity in findings between them, and we then coded and thematized, and created a lines-of-arguments synthesis based on analogous and refutational relationships between the themes in a back-and-forth process^21^, in which all three authors participated.

### Research ethics assessments

It is not necessary to apply to a research ethics committee for a project where qualitative meta-ethnography is used as a method^15^. The quality of the research ethics in each study was, however, appraised using the CASP instrument^20^.

## RESULTS

The findings across the seven included studies are presented with the help of an overarching theme, which is covered by the presentation of four main themes with their respective subthemes. Table 4 shows the developed overarching theme, the main themes and the subthemes, as well as which articles relate to them, respectively.

**Table 4:** Overarching theme, main themes and subthemes

*Overarching theme: To comprehend and to be understood as a unique person*				
**Main themes**	A desire to be met with respectful understanding	Violations and feelings of inferiority	Ethical and accurate interpreting	Caring midwives and new fellowships
**Subthemes**	Inadequate communication: a source of insecurity	Mutual loss of important information	Family and friends as interpreters	Caring and respect
				Continuity of care
	Participation in the delivery of information	Feeling stupid and treated accordingly	Professional interpreting services	
				Fellowship and increased language comprehension
	Basic health information	Perceived enforced consent		
		Avoidance behavior		
**References**	[12], [22], [23], [24], [25], [26], [27]	[12], [22], [23], [25], [26], [27]	[12], [22], [23], [25], [26], [27]	[12], [22], [23], [25], [26], [27]

The 142 women in the studies represented 42 different nationalities and were residents in 6 different host countries. The host countries represented were Canada, Finland, Norway, Sweden, Switzerland (represented twice), and the UK. The women originated from Albania, Australia, Bangladesh, Bolivia, Bosnia, Brazil, Burma, Chile, China, Columbia, El Salvador, Eritrea, Estonia, Ethiopia, Germany, Greece, Hungary, India, Iraq, Iran, Kosovo, Lebanon, Mauritania, Morocco, Pakistan, Peru, Philippines, Poland, Portugal, Russia, Slovenia, Spain, Sudan, Syria, Tajikistan, Thailand, The Dominican Republic, Turkey, Uganda, USA, Uzbekistan, and Vietnam, although one study did not specify the women’s nationality in their study^23^. It is not defined in the various included studies what degree of host-country linguistic skills the women possessed. The quotations used in the analysis process were where the women themselves had addressed a subjective issue related to the language.

### A desire to be met with respectful understanding

All included studies showed that women appreciated or expressed the need to be met with respectful understanding during the pregnancy, birth and postpartum follow-up^12,22-27^.

Both what was said and how this was conveyed were important. When communication was inadequate, it could create great insecurity among the women. They wanted to contribute to how and when the information was provided, or they had an opinion concerning what would have been an appropriate approach. Moreover, they sometimes needed information that the midwives mistakenly assumed they already knew^12,22-27^.

*Inadequate communication: a source of insecurity*

All the studies showed that inadequate communication was a source of insecurity^12,22-27^. When communication was inadequate, the women were placed in a vulnerable situation. The women wanted to understand their own situation, and were dependent on the midwives to acquire this understanding^12,22-27^.

The women wanted oral information and were dependent on the information being comprehensible for it to make sense and be reassuring. Both the speed of the midwives’ oral presentation and the midwives’ dialects could make it challenging to understand the midwives’ speech. For example, one of the women expressed that she felt it was inconsiderate of the midwife to speak quickly, even though the midwife knew that the woman did not know her language well and had a different mother tongue, and that this was a source of frustration for her^26^.

The women could also become frustrated if they were overwhelmed by too much information at once, or if, due to language difficulties, they received only a minimum of information. Both the women and the midwives tried to compensate for the lack of communication, but it was often insufficient. Examples include using pictures, gesturing, or showing emotions. Other approaches involved the women searching the internet themselves or trying to memorize a translation of something specific they wanted to bring up during a consultation. Personal, social and organizational factors could further exacerbate language difficulties – for example, it was difficult for some women to make appointments by telephone due to language barriers^24,25^. Some also felt that they were living at the mercy of the midwives in terms of service and follow-up. The women felt that the quality of healthcare that was provided and offered would be determined by that specific midwife^27^.

*Participation in the delivery of information*

The analysis showed that the women wanted information in an understandable language and in an appropriate way, preferably in their native language^22,24,26^. It was also stated that they wanted a presentation of the alternatives for how the information could be provided, and they wanted to be able to make a choice. Several women wanted both written and oral information, and wanted to be given the option of choosing the language of the presented information^22,24,26^. Some of the women also appreciated that the midwives were emotionally involved in their situation, instead of just giving what was necessary without showing any caring^26^.

*Basic health information*

Several studies showed that the women often lacked knowledge about the healthcare system of the host country, and they had a low level of health literacy^12,22-24,26^. Some of the midwives needed to explain and give information about subjects they had originally assumed the women would already have knowledge about^12,22-24,26^.

### Violations and feelings of inferiority

Several women addressed, in one form or another, a violation or misconduct resulting from language difficulties^12,22,23,25-27^. This was expressed either by the fact that there was a loss or potential loss of important information, or by actions or statements that made the women feel stupid. They could also experience shame, vulnerability, nervousness, tension, and mistrust, or they were subjected to a feeling of forced consent in dramatic situations^25,27^.

*Mutual loss of important information*

Communication difficulties could lead to mutual loss of information and additional needs^12,22,23,25-27^. Without a common language, the women experienced frustration, and perceived the relationship as superficial. Information, such as medical history that was relevant to the pregnancy, posed a risk when it was lost because of language issues. Moreover, things could occur during the pregnancy or during childbirth that the women did not understand or were able to express. Postpartum depression was mentioned as one such condition, where the midwives sometimes neither identified nor were informed about it.

When the women could not express their feelings or explain their situation, they risked experiencing that this part of the care was neglected, and therefore the focus was only on the necessary physical care. It is also mentioned that the midwives could be perceived as dominant or authoritarian because of inadequate communication, in that they seemed uninterested in the women’s experiences and perspectives^27^.

*Feeling stupid and being treated accordingly*

The women could struggle with negative emotions in their contact with midwives when their language skills were inadequate^12,22,23,25,27^. Feeling stupid and being treated as if they were stupid, was a recurring theme. They could also feel shame, vulnerability, nervousness, tension, and mistrust. The women described situations where they experienced a negative attitude towards their own abilities because of the language difficulties and the limitations that followed. This was perceived in one of the studies as very stressful and offensive to the women in question. Misplaced laughter could also be perceived as humiliating and hurtful^12^.

*Perceived forced consent*

Some women mentioned situations, especially acute ones that ended in perceived forced consent. The women felt that they did not understand because they were unable to obtain information and were therefore not able to give informed consent to interventions. This could also have serious consequences for the woman’s birth experience, as well as having an impact on her mental health. In some cases, the women felt mild concern about information they did not understand sufficiently, but in its most serious form, this could lead to existential anxiety and trauma^26,27^.

*Avoidance behaviour*

There were also women who, in addition to bad experiences, perceived these experiences as being of such a high degree of difficulty that, later in life, they avoided seeking care, unless it was necessary^25,27^. It is also stated that this avoidance behavior could lead to serious outcomes – for example, if the women postponed getting help and this affected their babies^25^.

### Ethical and accurate interpreting

Most of the studies showed that interpreters were used as a communication support, either by means of informal or professional interpreters with personal (or face-to-face) contact, or by telephone^12,22,23,25-27^.

*Family and friends as interpreters*

In the studies, some women spoke about the use of informal interpreters. Using family and friends as interpreters could be perceived as safe and familiar to the women, but was also mentioned as a possible cause of some ethical challenges when the women did not want to share sensitive information^22,26^. The use of relatives as interpreters occurred both because the women were not informed about their rights and the opportunity to use a professional interpreter, but also because their family or friends were available. Some of the women also stated that it was an active choice to use family or friends instead of a professional service, because it felt more comfortable^22^.

*Professional interpreting services*

The studies showed that the women felt dependent on the interpreters for understanding. At the same time, the situation with a professional service could be uncomfortable – especially in a very intimate situation such as childbirth^12,22,23,25-27^. The interpreters were also perceived as being inaccessible in several cases. The women did not know how to ask for the service, nor that this was a statutory right. Some women felt they would be a burden if they asked for an interpreter and felt that it could affect their relationship with health personnel negatively. There were also differences concerning the availability of the interpreters and in which situations the women perceived formal interpreting services as a good solution^12,26,27^. In maternity care, there were fewer concerns than during childbirth, and often a telephone interpreter was not presented as an alternative during childbirth because the birth was unpredictable and could just as easily happen at night. Furthermore, some of the women did not trust a stranger, although other women had no hesitations about using an interpreter because they only cared that what was said was translated correctly; it did not matter who it was as long as the translation was correct^22^.

### Caring midwives and new fellowships

The women pointed to their relationship with the midwives, continuity in the services, and social and structural factors as being relevant to their experience during pregnancy, childbirth, and the postpartum period^12,22,23,25-27^.

*Caring and respect*

The women said they appreciated the warmth and gestures that showed that the midwives cared^12,22,23,25-27^. Feeling that the midwives showed them individual consideration, as well as the creation of an atmosphere of calm and security, was described as positive. Individual considerations and gestures could involve simplifying their language for her, speaking more slowly in a respectful way, or responding caringly to what they were told. Being taken seriously was important for the women. Fewer complications were described when the women felt taken care of and listened to^25^.

*Continuity of care*

Some women mentioned how continuity and follow-up by the same midwife over time could provide great security and be an advantage, because the woman’s history was known and agreements were implemented as expected^12,22^. A lack of continuity could force the women to repeat their history several times and result in not having their requests and needs followed up^22^. It could also be a disadvantage if the continued relationship with the midwife was difficult^12^.

*Fellowship and increased language comprehension*

The studies showed that several women found fellowship and socialization in connection with birth preparation groups or integration programmes^12,23,25,26^. They experienced a sense of belonging and met others in the same situation, and this could help to strengthen their language skills and structural understanding of the host country, thereby enabling increased integration and preparation for birth. They could, for example, gain information, learn the language and societal structure, or even make friends. Some women took it for granted that they had personal responsibility for integration and for understanding. At the same time, being a recent arrival and thereby having the lowest degree of integration and understanding could be a burden^12^. Some also mentioned that the language barrier itself could be the factor that made them avoid the groups or programs that were intended to help them^26^.

### The synthesis

**Figure 2:**
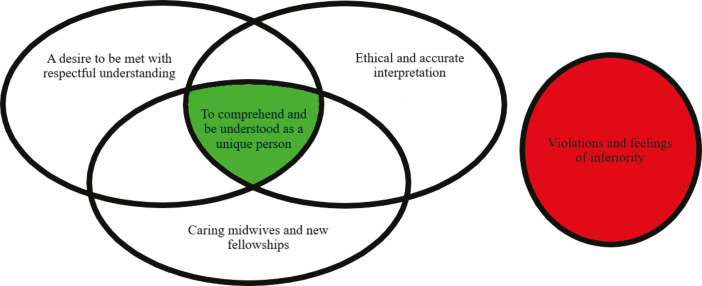
The lines-of-argument synthesis model ‘to comprehend and be understood as a unique person’

The model below (Figure 2) shows how the lines-of-argument synthesis^21^ is created by the analogous relationship between the three synthesized themes: ‘a desire to be met with respectful understanding’, ‘ethical and accurate interpreting’, and ‘caring midwives and new fellowships’. These have a core content that, by involving the various dimensions, enhances intercultural caring^9-13^ and minimizes the risk of violations and suffering related to care^14^. ‘Feeling violated and inferior’ is the theme that stands in a refutational relationship^21^ to the three other themes. Feeling violated and inferior means standing on the outside, feeling alone, and not feeling comprehended or understood.

Our meta-ethnographic translation and analysis process^21^ enabled us to develop the lines-of-argument synthesis of the analogous and refutational relationships between the themes, as visualized in the synthesized model (Figure 2). To the left is the core theme: ‘to comprehend and be understood’. This is created by three of the main themes that should be present in midwifery care when there are language challenges between the immigrant woman and the midwife. The risk of ‘violations and feelings of inferiority’ is the fourth main theme that stands in opposition to the core theme. Violations and feelings of inferiority related to language challenges in care can occur in isolation or in the intersection between individuals, but by implementing the core and its dimensions, the risk of suffering related to care^14^ may be reduced. The green color symbolizes immigrant women’s growing health and well-being, which is aimed for in midwifery care, versus the red stop sign that represents an encounter where the language remains a challenge and may lead to violation and inferiority, which taxes a woman’s health. The model was developed to visualize the uniqueness of findings, namely that the experiences of the immigrant women are of central importance.

‘To comprehend and be understood’ is about finding the approach that allows the woman precisely this experience. The women who feel cared for in this way do not show signs of having experienced violation or suffering related to care^14^ in those circumstances. Furthermore, it concerns reaching the core in all the dimensions that have emerged during the synthesis as violations and suffering related to care^14^ could occur under the three individual dimensions in the model but were not described in those cases where all were equally considered. ‘A desire to be met with respect’ involves information participation and the need for basic health information where midwives must ensure that the women have knowledge of the host country’s service structure and knowledge of their own body and health, without taking anything for granted. ‘Ethical and accurate interpreting’, where the key is to establish interpretation, concerns circumstances where the woman herself experiences adequate translation. ‘Caring midwives and new fellowships’ refers to those cases where care and respect, continuity, socialization and increased language comprehension are of central importance. It is important to point out that fellowship and socialization, followed by an increased language comprehension and the creation of meaning for women, does not involve immigrant women in any way rejecting their own culture or values.

## DISCUSSION

This meta-ethnography synthesizes immigrant women’s experiences of language challenges during pregnancy, birth and the postpartum period in a new country. We collected articles with subjective narratives from immigrant women from several countries. There may be differences in the attitude, system, structure, integration policy or social apparatus surrounding the expectant mother that are relevant as part of the immigrant woman’s experiences. By following the principles of eMERGe^17^, we nevertheless tried to provide a high enough degree of transparency and reflexivity so that the insight into the process lends reliability to the research.

The synthesis show ‘to comprehend and be understood as a unique person’ as the main finding from the immigrant women’s perspectives when language challenges could be resolved. The new understanding is based on three themes that show that women wanted to be met with respectful understanding, they needed ethical and accurate interpreting, and they hoped for and described the meaning of encountering caring midwives and new fellowships. A fourth theme, ‘violations and feeling of inferiority’ shows the opposite experience, where the language challenges remain and cause an extra burden in care, resulting in suffering related to care for the immigrant women^14^. We use the theories of intercultural caring^9^ and suffering related to care^14^ in order to deepen the understanding of the new synthesis model (Figure 2).

### Intercultural caring

Being confirmed is a universal need, regardless of language issues. As human beings, this is our most basic emotional need, and this need is fundamental in all healthcare practice^28^. Wikberg’s theory of intercultural caring as the theoretical perspective^9^, involves the preservation of four dimensions: the universal, the cultural, the contextual, and unique care. One approach for including the dimensions of care was through interest in the woman’s culture, where the midwife asked what needs she had and what she wanted to be considered based on her culture^10,13^. If the midwife showed an interest in both the woman, her culture and her wishes, and made her feel that she was being taken care of, all four dimensions of Wikberg’s theory of intercultural caring would be taken into account^9^. According to this theory, it was only when all four dimensions have been considered that the midwife would ensure that the woman received the individual care she needed, and that the risk of violations would be minimized. Unlike Wikberg^9^, Leininger^29^ focused on the differences and emphasized the importance of learning about and understanding several aspects of different cultures, ideally seeking to take their perspective in order to provide good care. Other studies have shown that women appreciated it when the midwives acted in a culturally sensitive manner, without the women feeling a need for the midwives to immerse themselves too deeply in their culture and customs^12,30,31^.

All the included studies show that a holistic understanding in the pregnancy follow-up was crucial for the immigrant women to feel safe^12,22-27^. This is supported in Wikberg’s theory^9^ by means of a description of the unique aspect of intercultural caring, where communication is of utmost importance. The information provided should be given in both written and oral form, and then in an understandable language^22,24,26^. That health information should be given verbally by health personnel is confirmed by other studies^32-34^. Another study shows one should avoid medical professional language when talking to these women^41^. Our findings also show that several of the women in the primary studies^22,24,26^ wanted written information in an understandable language. Other studies^32,35^ point out limited translated written material in maternity care. Another study^35^ shows that it is often problematic for women to utilize the material even if it has been translated, because their health knowledge is at such a level that they need an oral explanation from the midwife to utilize the information. The problematic level of knowledge also emerged in our analysis, where women may lack knowledge about their bodies and general health issues but also about structures, systems and the health service’s purpose and availability. This issue is confirmed by several articles^32,34,36,37^. One study points to the women’s pre-immigration experiences playing a major role in whether and how they relate to the healthcare system and health services^36^. The results indicate that birth preparation groups or integration programs could contribute to increased independence by strengthening the language and the understanding of the culture of the host country. The birth preparation groups provided support in pregnancy and an increased preparation for childbirth and the postnatal period^12,23,25,26,37^. Both our and other studies also point out that language difficulties could result in women stopping attending such courses, namely due to language difficulties^26,38,39^, which is understandable, but potentially unfortunate for both the women and the health professionals in contact with the women. Through such courses, the universal and the contextual care will be able to be present^9^.

### Suffering related to care

Suffering could be inflicted on women in the maternity care setting^14^. This occurred unintentionally on the part of the midwives, but was an unfortunate result of a care situation that was characterized by the caregivers’ insufficient knowledge and lack of reflection on the situation with regard to language challenges^14^. Midwives were often under the impression that gestures and images were sufficient, but misunderstandings could go unnoticed and information could become totally overwhelming for the women^27^. The women sought information on their own, through the internet, in order to feel prepared for childbirth and they felt that they had to learn about the health system on their own. Women with language challenges could benefit from computer and internet skills for seeking information^36^, but when women experienced having to look up information on their own and did not gain sufficient knowledge to meet their situation in the healthcare system, it is obvious that there was a failure at all four levels of caring in Wikberg’s theory^9^. The midwife could also overestimate the woman’s language skills if they were able to communicate in a common language. Even in cases where the women were skilled in several languages, a stressful situation could lead them to needing to speak their native language, which made communication difficult^40^. There could also be situations where the women pretended to understand in order to be left alone because they were unable to explain or understand and therefore resigned themselves to not understanding^26^. When the women pretended to understand, important information could be lost, and midwives failed to secure this. Midwives could further misinterpret the woman’s silence as a sign that things were going well and that the woman was in control. This could result in a negative encounter that could lead to suffering related to care^14^. A negative encounter could prevent the growth and development of the relationship between the woman and the midwife and could also make the situation difficult for the immigrant woman^14^. In the event of perceived suffering related to care, it would be impossible for the midwife to implement all of Wikberg’s four dimensions of intercultural caring^9^. Language barriers could, in the worst case, lead to violations and perceived forced consent in emergency situations, and could have serious consequences for the women, because they were not able to fully understand the information provided^26,27^. The women could also feel stressed and ashamed of their inability to communicate^22^. The experience of being ridiculed and feeling stupid also appeared in several articles^26,27,38,41^. Suffering related to care^14^ could also occur when the women did not have enough information to understand the healthcare system in the host country, when they had low health literacy, or when they did not understand the care or interventions^12,22-24,26,27^. The women’s level of health literacy affected their ability to cope during pregnancy, birth and the postnatal period^23^. The women highlighted the need for information about the healthcare system in the host country, which was sometimes taken for granted by the health personnel^12,22^. Higher health competence among immigrants could lead to a greater understanding of the importance of pregnancy follow-up and could lead to a better maternity care, whilst a lower level of health literacy could lead to poorer attendance as the women were not aware of the importance of check-ups^36^. When midwives have knowledge of the contextual aspects of the women’s situations, they should be able to make the women familiar with the information they need^9,13^.

Interpreting situation with relatives can become problematic as shown in our study and cause stressful situations for the people involved^36^. Such situations are highly undesirable, and every effort should be made to avoid these occurring. Wikberg^13^ makes it clear in her theory that a professional interpreting service is often needed in order to establish a satisfactory relationship between the midwife and the expectant mother. The importance of a well-functioning interpreting service in maternity care is also emphasized in several other studies^10,32,33,35,36,42-45^. As we have shown in the results, there is variation in whether the women feel the need for an interpreter. One study^45^ points out that a person may experience difficulties with a secondary language in a stressful situation such as birth, even if they speak the language fluently. Stressful situations can therefore make it more problematic for the woman to communicate and understand. The same can apply to the use of medical terminology. There are several potential consequences of a lack of an interpreter, as our findings also show. Enforced consent and the traumatization of the women are the worst-case scenarios of not having a communication flow that works^45^. It is further pointed out that the provision of an interpreting service is cost-saving in the long-term, which could be an important factor to explain to women who may feel that they are burdening the system by bothering to need an interpreter^45^.

## CONCLUSIONS

The results showed that immigrant women who have language difficulties in the host country were a vulnerable group who were at risk of experiencing suffering related to care^14^ and feeling violated due to linguistic difficulties during pregnancy, childbirth, and the postnatal period. In our meta-ethnography, these violations were related to a lack of consent, avoidance behavior and mutual loss of information, which could lead to difficult emotions. A synthesized model was developed to show which aspects the study revealed as being central to the promotion of intercultural caring and mutual understanding in midwifery care for immigrant women.

## Data Availability

Data sharing is not applicable to this article as no new data were created.
